# Instituting Sustainable Geriatric Care in Africa: The Roles of Sociocultural Constructs

**DOI:** 10.24248/EAHRJ-D-19-00020

**Published:** 2019-07-30

**Authors:** Jude T Ssensamba, Dennis M Ssemakula, Jake MacLeod, Justine N Bukenya

**Affiliations:** a Department of Community Health, College of Health Sciences, School of Public Health, Makerere University, Kampala, Uganda; b Center for Innovations in Health Africa, Kampala, Uganda; c University of Edinburgh, Edinburgh, UK

## Abstract

The demographic shift in Africa is seeing more people make it to old age (60 years or over), a state associated with an increased risk of acquiring communicable and non-communicable diseases, and demand for specialised health care. With many African health systems still struggling with infectious diseases, inadequate funding, poor infrastructure and lack of skilled human resource for health, how best can they provide quality, sustainable geriatric care services to their ageing population? This commentary highlights “Africa's social-cultural structure” as an opportunity health policy makers could tap into, to design patient-centred, sustainable, inexpensive, and socially acceptable geriatric interventions.

## INTRODUCTION

**FIGURE 1.**
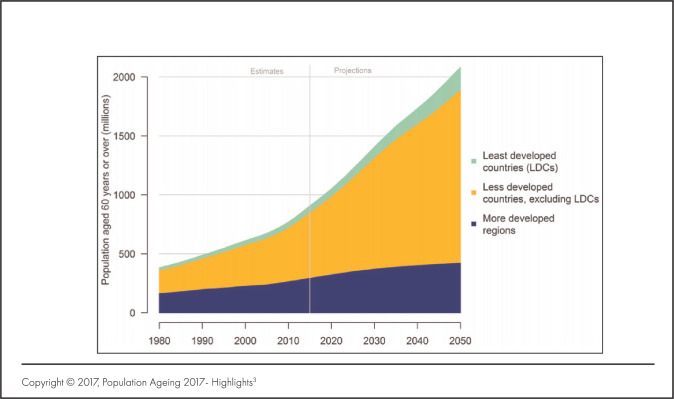
Number of Persons 60 Years and Over by Development Group (1980-2050)

With an improvement in the quality of life, security and health services, Africa is experiencing a demographic shiff. This transition comes with a rising number of old persons (people above 60 years). By 2050, the world will be home to 2.1 billion elderly people, with over 76% of them living in the developing world (Figure 1). Africa expects more than 10% of its population to be aged by then.^1^ To highlight the urgency for action, in 2016, the World Health Organisation (WHO) and member states through the sixty-ninth World Health Assembly, passed resolution 69.3. This resolution formed the backbone of the WHO's global strategy and plan of action on ageing and health, whose objectives were to call on all member governments to institute sustainable policies and measures that promote the wellbeing and health of old persons.^2^

**FIGURE 2.**
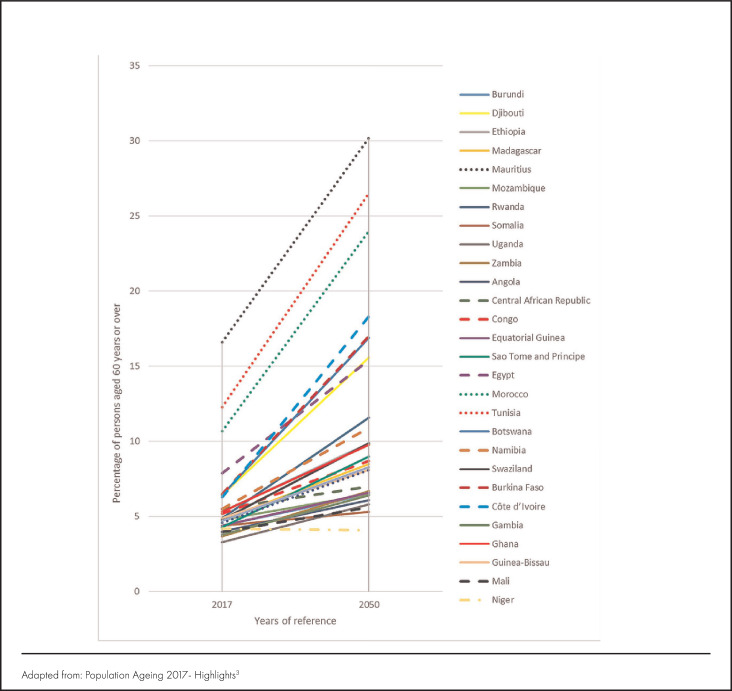
Percentage of Persons 60 Years and Over Across Select African Countries (2017-2050)

Adapted from: Population Ageing 2017-Highlights^3^

However, how prepared is developing Africa that continues to struggle with the burden of infectious diseases to face its next public health challenge of ageing, and what strategies are countries in Africa leveraging to ensure healthy ageing for old persons?^4,5^ In the developed world, the more than a century old geriatrics specialisation has metamorphosed into a complex practice, and residential and community-based care models for supporting old persons to live healthy and productive lives have been developed.^6-8^ On the other hand, there is little knowledge and information on how geriatric care and support can be best established in Africa's health systems, where the record number of people living to at least 60 years of age is predicted to continue increasing for at least the next 50 years (Figure 2).

Based on previous health programming experiences, the easiest alternative would be to directly replicate already existing western models like building geriatric homes and setting up long-term care insurance programmes like what is practised in Japan.^9^ However, this would imply that Africa is unaware of the social-cultural, logistical and long-term economic implications of setting up such systems in countries already struggling with poor health infrastructure, inadequate equipment and logistics, and inadequate skilled human resource for health.^10-13^

## COULD AFRICA'S SOCIOCULTURAL STRUCTURES BE THE GAME-CHANGER?

Although the influence of Westernisation and globalisation continue to take root in Africa, the continent is known and continues to thrive on strong community-based sociocultural structures. Herein, relatives of different ages and generations, neighbours and friends tend to live together in communities, sharing common social services and resources, supporting each other, and making collective decisions regarding their health and other social obligations.^14-17^ This experience is stronger in rural settings, home to the majority of old persons, who culturally are the central pillars of such communities, are held in high esteem, and all community members work towards their wellbeing.^14,18^ The question is, could this paradigm provide a framework for geriatric researchers to come up with sustainable Community-Based Geriatric Care (CBGC) models for Africa?

## SUPPORTING THE ELDERLY IN AFRICA IS A TRANSGENERATIONAL COMMUNAL ROLE

**FIGURE 3.**
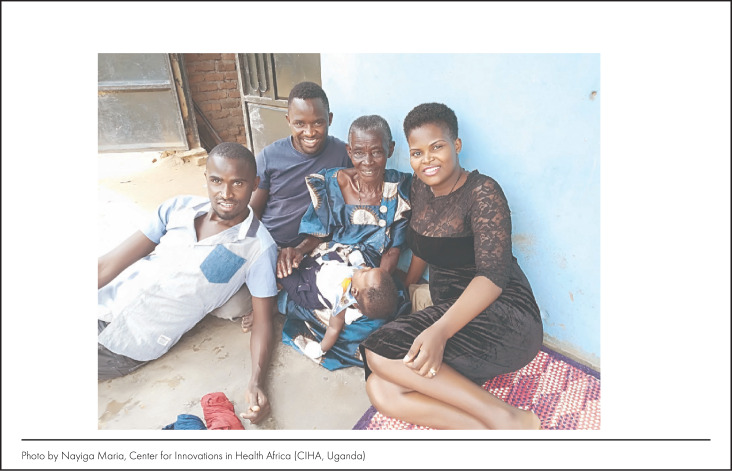
Supporting the Elderly in Africa is a Transgenerational Communal Role

Community or home-based geriatric care models have successfully improved health outcomes of the elderly in the developed world, tailored on system approaches where geriatricians, doctors, nurses, social care workers, and volunteers visit older adults in homes to provide nursing, psychosocial and home support services.^7,19^ On the other hand, although there are no published works on CBGC in Africa's context, anecdotal evidence suggests an already existing but unexplored “community-led elderly care” model, where relatives and friends are the primary players tending to the health, personal, and psychosocial needs of the elderly (Figure 3). For example, it is common to find children, grandchildren, relatives, friends, and neighbours reminding the elderly when to take their medicines and taking them out for walks and other physical engagements.

Photo by Nayiga Maria, Center for Innovations in Health Africa (CIHA, Uganda)

**FIGURE 4.**
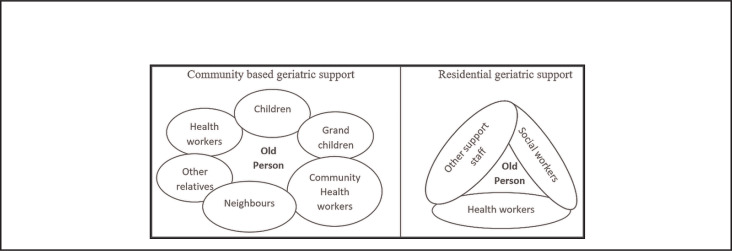
Key Players in Community-based Versus Residential-based Geriatric Support

Tailoring geriatric care to Africa's existing social-cultural context is more likely to be acceptable and sustainable, given that it avails an opportunity for patient-centred care, where family and community members directly take part in caring for their loved ones, thus minimising costs of care, and psychological effects like depression, already known to be prevalent among old persons staying in geriatric homes.^7,20,21^ Re latedly, with human resources for health in Africa continuing to be inadequate, training relatives, neighbours and friends in basic community based geriatric care would provide a viable alternative, given that engaging community based health volunteers with no medical background has been documented to help reduce childhood mortality and morbidity, and improve postnatal care and HIV care services.^22-24^

That said, CBGC models for Africa should be cognizant of and fit into its changing dynamics with regard to rural-urban migration, urbanisation, and socioeconomic transformation. There is also a lot to learn from the developed world with regard to planning for, and mitigating challenges such as physical stress on the caregivers, economic losses associated with time lost in providing such care, and most importantly setting up quality monitoring and assurance systems for better geriatric care delivery.

As geriatric professionals and health policy makers continue to contemplate the best strategies for instituting geriatric care in developing Africa, it is our view that these existing social norms and practices are explored as possible key pillars for designing socially, culturally acceptable, and economically sound geriatric care models.

## References

[1] UNFPA and HelpAge International. Ageing in the Twenty-First Century: A Celebration and A Challenge. New York: UNFPA and HelpAge International; 2012. https://www.unfpa.org/publications/ageing-twenty-first-century. Accessed 5 August 2019.

[2] World Health Organization (WHO). The Global Strategy and Action Plan on Ageing and Health. Geneva: WHO; 2017. https://www.who.int/ageing/global-strategy/en/. Accessed 5 August 2019.

[3] United Nations, Department of Economic and Social Affairs, Population Division. World Population Ageing 2017 - Highlights (ST/ESA/SER.A/397). New York: United Nations; 2017. https://www.un.org/en/development/desa/population/publications/pdf/ageing/WPA2017_Highlights.pdf. Accessed 5 August 2019.

[4] Murray CJ, Vos T, Lozano R, et al. Disability-adjusted life years (DALYs) for 291 diseases and injuries in 21 regions, 1990-2010: a systematic analysis for the Global Burden of Disease Study 2010. Lancet. 2012;380(9859):2197-2223. 10.1016/S0140-6736(12)61689-4. Medline 23245608

[5] Tout K. Elderly Care: A World Perspective. London: Chapman & Hall; 1993. 10.1007/978-1-4899-4509-9

[6] Ausserhofer D, Deschodt M, De Geest S, et al. “There's No Place Like Home”: a scoping review on the impact of homelike residential care models on resident-, family-, and staff-related outcomes. J Am Med Dir Assoc. 2016;17(8):685-693. 10.1016/j.jamda.2016.03.009. Medline 27130574

[7] Bulsara C, Etherton-Beer C, Saunders R. Models for community based day care for older people: a narrative review. Cogent Soc Sci. 2016;2(1):1267301. 10.1080/23311886.2016.1267301

[8] Béland F, Hollander MJ. Integrated models of care delivery for the frail elderly: international perspectives. Gac Sanit. 2011;25 Suppl 2:138-146. 10.1016/j.gaceta.2011.09.003. Medline 22088903

[9] Okamoto Y. Health care for the elderly in Japan: medicine and welfare in an aging society facing a crisis in long term care. BMJ. 1992;305(6850):403-405. 10.1136/bmj.305.6850.403. Medline 1392924 PMC1883155

[10] Mayosi BM, Lawn JE, van Niekerk A, et al. Health in South Africa: changes and challenges since 2009. Lancet. 2012;380(9858):2029-2043. 10.1016/S0140-6736(12)61814-5. Medline 23201214

[11] Maher D, Smeeth L, Sekajugo J. Health transition in Africa: practical policy proposals for primary care. Bull World Health Organ. 2010;88(12):943-948. 10.2471/BLT.10.077891. Medline 21124720 PMC2995191

[12] Lagomarsino G, Garabrant A, Adyas A, Muga R, Otoo N. Moving towards universal health coverage: health insurance reforms in nine developing countries in Africa and Asia. Lancet. 2012;380(9845):933-943. 10.1016/S0140-6736(12)61147-7. Medline 22959390

[13] Samb B, Desai N, Nishtar S, et al. Prevention and management of chronic disease: a litmus test for health-systems strengthening in low-income and middle-income countries. Lancet. 2010;376(9754):1785-1797. 10.1016/S0140-6736(10)61353-0. Medline 21074253

[14] Fiske AP. Structures of Social Life – The Four Elementary Forms Of Human Relations: Communal Sharing, Authority Ranking, Equality Matching, Market Pricing. New York: Free Press; 1991.

[15] Arowolo D. The effects of Western civilisation and culture on Africa. Afro Asian J Soc Sci. 2010;1(1).

[16] Peil M. Family help for the elderly in Africa: a comparative assessment. South Afr J Gerontol. 1995;4(2):26-32. 10.21504/sajg.v4i2.83

[17] Cassiman A. Home call: Absence, presence and migration in rural northern Ghana. Afr Identities. 2010;8(1):21-40. 10.1080/14725840903438269

[18] World Health Organization (WHO). Towards Long-Term Care Systems in sub-Saharan Africa. Geneva: WHO; 2017. https://www.who.int/ageing/publications/ltc-series-subsaharan-africa/en/. Accessed 5 August 2019.

[19] Siegler EL, Lama SD, Knight MG, Laureano E, Reid MC. Community-based supports and services for older adults: a primer for clinicians. J Geriatr. 2015;2015:678625. 10.1155/2015/678625. Medline 25729774 PMC4339950

[20] McDougall FA, Matthews FE, Kvaal K, Dewey ME, Brayne C. Prevalence and symptomatology of depression in older people living in institutions in England and Wales. Age Ageing. 2007;36(5):562-568. 10.1093/ageing/afm111. Medline 17913759

[21] Ames D. Depressive disorders among elderly people in long-term institutional care. Aust N Z J Psychiatry. 1993;27(3):379-391. 10.3109/00048679309075793. Medline 8250780

[22] Brenner JL, Kabakyenga J, Kyomuhangi T, et al. Can volunteer community health workers decrease child morbidity and mortality in southwestern Uganda? An impact evaluation. PLoS One. 2011;6(12):e27997. 10.1371/journal.pone.0027997. Medline 22194801 PMC3237430

[23] Namukwaya Z, Barlow-Mosha L, Mudiope P, et al. Use of peers, community lay persons and Village Health Team (VHT) members improves six-week postnatal clinic (PNC) follow-up and early infant HIV diagnosis (EID) in urban and rural health units in Uganda: a one-year implementation study. BMC Health Serv Res. 2015;15:555. 10.1186/s12913-015-1213-5. Medline 26666331 PMC4678627

[24] Chang LW, Kagaayi J, Nakigozi G, et al. Effect of peer health workers on AIDS care in Rakai, Uganda: a cluster-randomized trial. PLoS One. 2010;5(6):e10923. 10.1371/journal.pone.0010923. Medline 20532194 PMC2880005

